# Modulatory effects of nitric oxide-active drugs on the anticonvulsant activity of lamotrigine in an experimental model of partial complex epilepsy in the rat

**DOI:** 10.1186/1471-2202-8-47

**Published:** 2007-07-03

**Authors:** Pierangelo Sardo, Giuseppe Ferraro

**Affiliations:** 1 Dipartimento di Medicina sperimentale, Sezione di Fisiologia umana "G. Pagano", Università degli Studi di Palermo, C.so Tukory, 129 – 90134 Palermo, Italy

## Abstract

**Background:**

The effects induced by administering the anticonvulsant lamotrigine, the preferential inhibitor of neuronal nitric oxide synthase 7-nitroindazole and the precursor of NO synthesis L-arginine, alone or in combination, on an experimental model of partial complex seizures (maximal dentate gyrus activation) were studied in urethane anaesthetized rats. The epileptic activity of the dentate gyrus was obtained through the repetitive stimulation of the angular bundle and maximal dentate gyrus activation latency, duration and post-stimulus afterdischarge duration were evaluated.

**Results:**

Either Lamotrigine (10 mg kg^-1^) or 7-nitroindazole (75 mg kg^-1^) i.p. administration had an anticonvulsant effect, significantly reducing the number of animals responding to angular bundle stimulation. On the contrary, i.p. injection of L-arginine (1 g kg^-1^) induced an aggravation of the epileptiform phenomena, demonstrated by the significant augmentation of the duration of both maximal dentate activation and afterdischarge. Furthermore, the injection of lamotrigine and 7-nitroindazole in combination significantly increased the anticonvulsant effects induced by the same drugs separately, either reducing the number of responding animals or decreasing both maximal dentate gyrus activation and afterdischarge durations. On the contrary, the combined treatment with L-arginine and lamotrigine did not modify the maximal dentate gyrus activation parameters suggesting an adversative effect of L-arginine-increased nitric oxide levels on the lamotrigine-induced anticonvulsant action.

**Conclusion:**

The present results indicate that the nitrergic neurotransmission exerts a significant modulatory role in the control of the development of paroxystic phenomena in the maximal dentate gyrus activation model of epilepsy. Finally, our data suggest a functional relationship between the nitric oxide system and the anticonvulsant effect of lamotrigine which could be enhanced by reducing nitric oxide levels and, conversely, dampened by an increased nitrergic activity.

## Background

Nitric oxide (NO) is a gaseous messenger synthesised from the oxidation of L-arginine by three different isoforms of NO synthase (NOS): the neuronal (nNOS) and endothelial isoforms are calcium activated, on the contrary the inducible isoform is activated by a calcium independent enzyme. NO acts on the soluble guanylyl cyclase (sGC), increasing the cellular concentration of cyclic guanosine monophosphate (cGMP), which is able to modulate several cellular functions as normal and pathological excitability, neuronal plasticity etc. [1]. In the central nervous system (CNS), NO acts as unconventional neurotransmitter; in fact, it contributes to the release of other neurotransmitters (e.g. glutamate, GABA, dopamine etc.), and participates to the synaptic plasticity, axonal elongation and other cellular functions [2]. Furthermore, NO has been involved in several neurological disorders as ischemia, trauma, neurodegenerative diseases etc., showing a particular functional relevance in the pathophysiology of neurotoxic and neuroprotective processes [3].

Within the disorders of CNS, NO has been also considered to play a fundamental role in the genesis and the spreading of the epileptiform hyperactivity [4]. In particular, several experimental researches have demonstrated the functional involvement of NO in both pro-convulsant and anticonvulsant phenomena but no definitive conclusions are still available [5,6]. Such heterogeneity of the responses to the pharmacological manipulation of the NO system could be related to the different models of experimental epilepsy used [7]. Furthermore, some excitatory effects could be attributed to the modifications of the cerebral blood flow induced by changes of NO levels [8,9]. Moreover, the functional interaction between NO and glutamate systems has been considered as a further possible source of the cited variability. In fact, NO is able to interact with the redox site of the N-methyl-D-aspartate (NMDA) receptor to glutamate, decreasing the responsiveness to glutamate agonists, particularly in all the conditions characterised by an "overactivity" of the glutamate receptor complex [10-13]. On the other hand, an abnormal increase in the activation of NMDA and non-NMDA receptors, as shown in the epileptogenesis and/or in the excitotoxic phenomena, is strictly linked to the production of NO and/or its related molecules [3]. Finally, it has been hypothesised that glial cells could constitute a further source of NO which exerts a neuroprotective action against NMDA-induced neurotoxicity [14]. In the last decade several researches have evaluated the interaction between the nitrergic system and some antiepileptic drugs (AEDs) with the aim to increase the efficacy of the anticonvulsant therapy. The activity of different AEDs seems to be strictly linked to a significant reduction of nNOS activity [15-18]. Interestingly, several experimental data have demonstrated the existence of a functional interaction between the second generation anticonvulsant lamotrigine (LTG) and the nitrergic system, although the observed effects are not univocal. The action of LTG, which shows a particular efficacy in human partial epilepsy, is characterised by the blockade of sodium voltage-gated channels, reducing high frequency firing of somatic action potentials and decreasing an excessive, potentially neurotoxic, glutamate release [19-21]. Furthermore, LTG is able to reduce NO release but, in the mouse model of maximal electroshock seizure, the combination between 7-NI and LTG was neutral in relation to a possible increase of the anticonvulsant effect [22,23]. On the contrary, it has been proposed that NO-mediated mechanisms are involved in the anticonvulsant efficacy of LTG [24].

In the present study, with the aim to clarify the functional interaction between the nitrergic neurotransmission and the LTG-induced anticonvulsant effect, we used an experimental model of partial complex epilepsy, the maximal dentate gyrus activation (MDA) which reproduces a common human epilepsy [25]. In particular, we have examined the effects of the anticonvulsant LTG administered alone or in combination with drugs modulating the NO neurotransmission. We have modified the level of endogenous NO through the administration of 7-Nitroindazole (7-NI), a preferential inhibitor of neuronal NOS using a dose (75 mg kg^-1^) which is able to reduce in several brain areas the nNOS activity within the range of 60 and 80% [26] and L-arginine, a precursor of the synthesis of NO. The time of onset and the duration of the ictal events were evaluated together with the analysis of the characteristics of dentate gyrus (DG) evoked responses to angular bundle (AB) stimulation.

## Results

**Figure 1:**
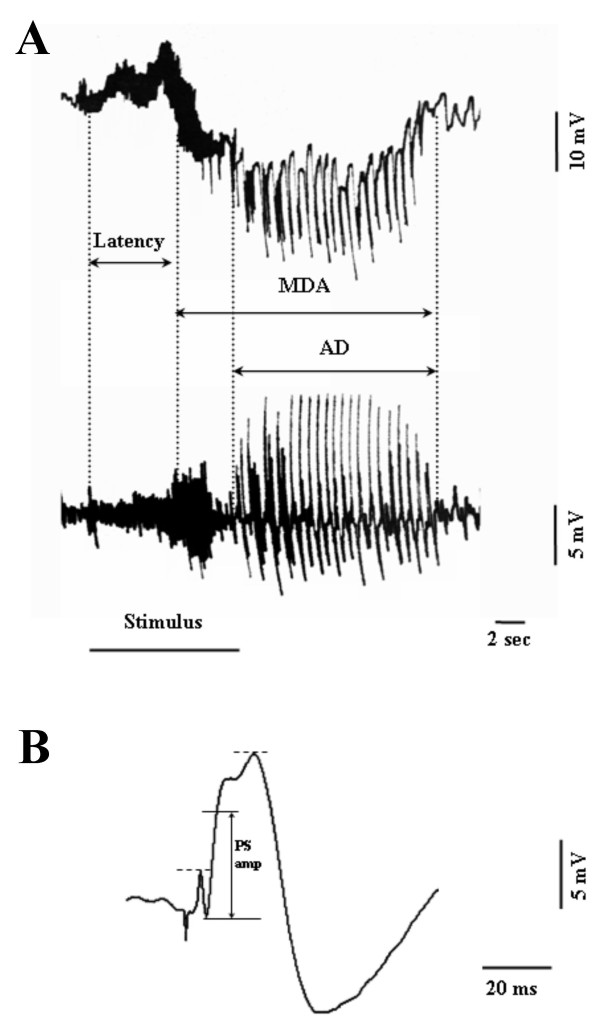
**Maximal dentate gyrus activation and evoked population spike parameters**. **A**: Measurements of latency and duration of maximal dentate gyrus activation (MDA) and afterdischarge (AD) during and after a 400 μA, 20 Hz stimulus train of the angular bundle (AB) for 10 sec. The recordings were amplified using low level DC (A) and a wide band AC (B) pre-amplifiers respectively. **B**: An average of five evoked responses in the dentate gyrus to the stimulation of the ipsilateral angular bundle. The amplitude of the population spikes (PS amp) was measured as illustrated in the figure, considering the mid-point between the dashed lines as the top of the PS. Calibration are indicated on the figure.

Once the MDA was elicited, the values for all the following parameters of the MDA were analysed: i) the onset of the MDA was considered as the time from the beginning of AB stimulation to the midpoint of the shift of the DC potential; ii) the total duration of the MDA was measured from the midpoint of the shift of DC the potential to the point at which the evoked paroxystic activity abruptly ceased; iii) the afterdischarge (AD) duration was measured from the end of AB stimulation to the end of the epileptiform activity (Figure 1A). The comparisons of the above cited parameters in DC- and AC-coupled traces did not show any significant difference Figure 1A).

The time course of MDA parameters (onset, MDA and AD durations) in control animals (n = 10) was monitored for at least 2 hours after the threshold intensity of stimulation was attained. The repetitive stimulations were not able to significantly alter the MDA parameters during all the observation period. Furthermore, in another group of animals i.p. injection of an adequate volume of vehicle (Dimethylsulfoxide - DMSO or saline) (n = 5 rats of each vehicle) did not cause any sort of modification of the three MDA parameters along the following 120 min of experimental observation.

### Effects of 7-Nitroindazole, L-arginine and LTG administration alone and in combination on the number of responding animals

**Figure 2:**
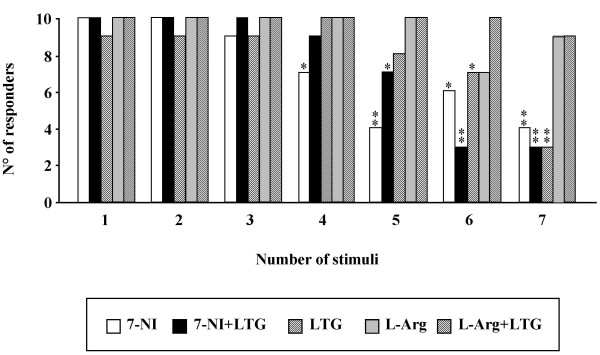
**Effects of 7-Nitroindazole, L-arginine and Lamotrigine administration alone and in combination on the number of responding animals**. The bars indicate the number of animals responding with a typical MDA activity to the angular bundle stimulation after each drug treatment. In abscissa the progressive number of train of stimuli applied every 10 min is indicated. The chi-square test was used to compare the animals responding and not responding to the electrical stimulation. The differences were considered marginally and highly significant at the level of P < 0.05 (*) and P < 0.005 (**) respectively.

The administration of 7-NI caused a reduction in the number of animals responding with MDA to the stimulation, starting 40 min after the drug administration with a maximum at the 50^th ^– 70^th ^min (-60 %, chi square = 8.571, DF = 1, P = 0.0034). Furthermore, the systemic administration of LTG induced a significant reduction in the number of animals responding to the stimulation sessions, starting at the 60^th ^min with a maximum 70 min after the LTG administration (-70 %, chi square = 10.769, DF = 1, P = 0.001). A significant and more prolonged decrease in the number of animals responding to the stimulations was also observed after 7-NI and LTG co-administration, starting at the 50^th ^min after drugs administration and maintained along the observation period, with a maximum at 60^th ^and 70^th ^min (-70 %, chi square = 10.769, DF = 1, P = 0.001). Moreover, no significant variation in the number of animals responding to the stimulation was noted under L-arginine alone or in combination with LTG. The Figure 2 shows all the variations in the number of responding animals for each pharmacological treatment.

### Effect of 7-Nitroindazole, L-Arginine and LTG administration alone and in combination on time of onset, MDA and AD duration

**Figure 3:**
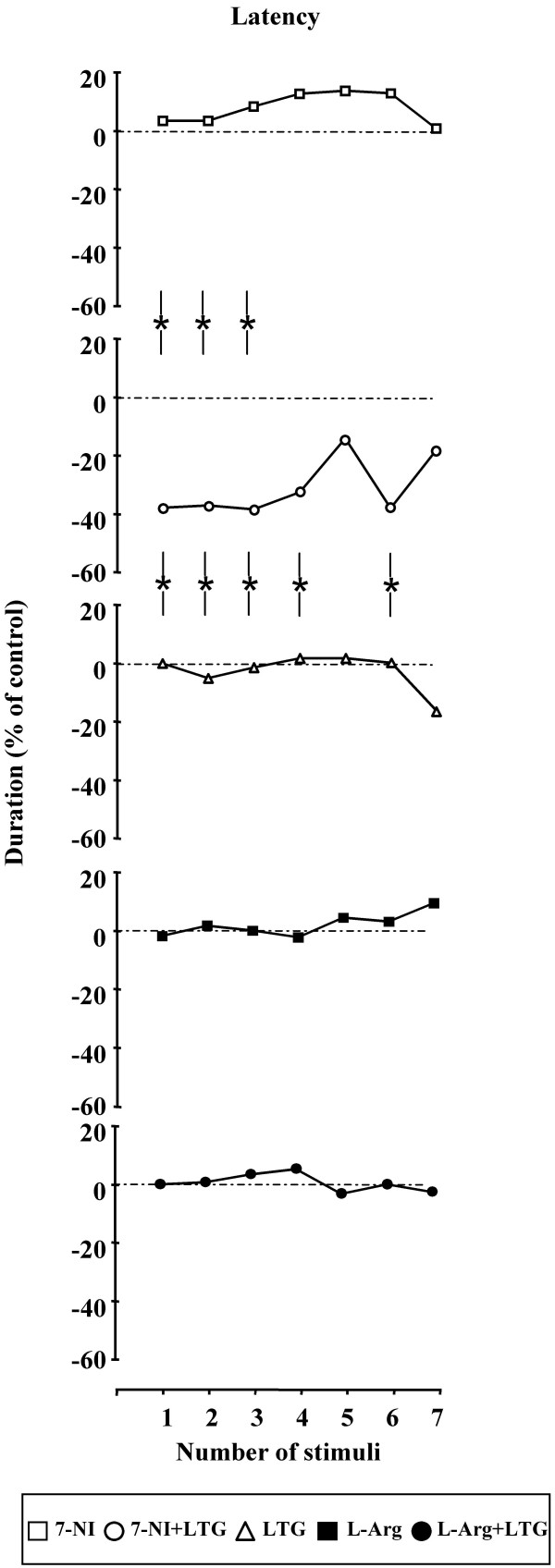
**Effects of 7-Nitroindazole, L-arginine and Lamotrigine administration alone and in combination on MDA latency**. Effects of 7-Nitroindazole (75 mg kg^-1 ^i.p.), L-arginine (1 g kg^-1 ^i.p.) and Lamotrigine (10 mg kg^-1 ^i.p.) administration alone and in combination on MDA latency (n = 10 rats for each treatment). In abscissa the progressive number of train of stimuli applied every 10 min is indicated. Asterisks between lines (-*-) indicate a significant difference between the two treatments (P < 0.05).

A) Latency (Figure 3) – 7-NI administration caused a moderate, not statistically significant increase of MDA latency with a maximum 50 min after drug injection. Furthermore, the co-administration of 7-NI and LTG caused a not significant decrease of MDA time of onset, more marked in the first 40 min after the drug treatment. LTG and L-arginine alone or in combination failed to induce significant modifications in MDA time of onset. When comparing the effects induced by 7-NI alone and in combination with LTG a statistically significant difference was highlighted during the first 30 min after drug administration. Furthermore, statistically significant differences were evidenced between the effects induced by LTG alone and in combination with 7-NI along all the observation period.

**Figure 4:**
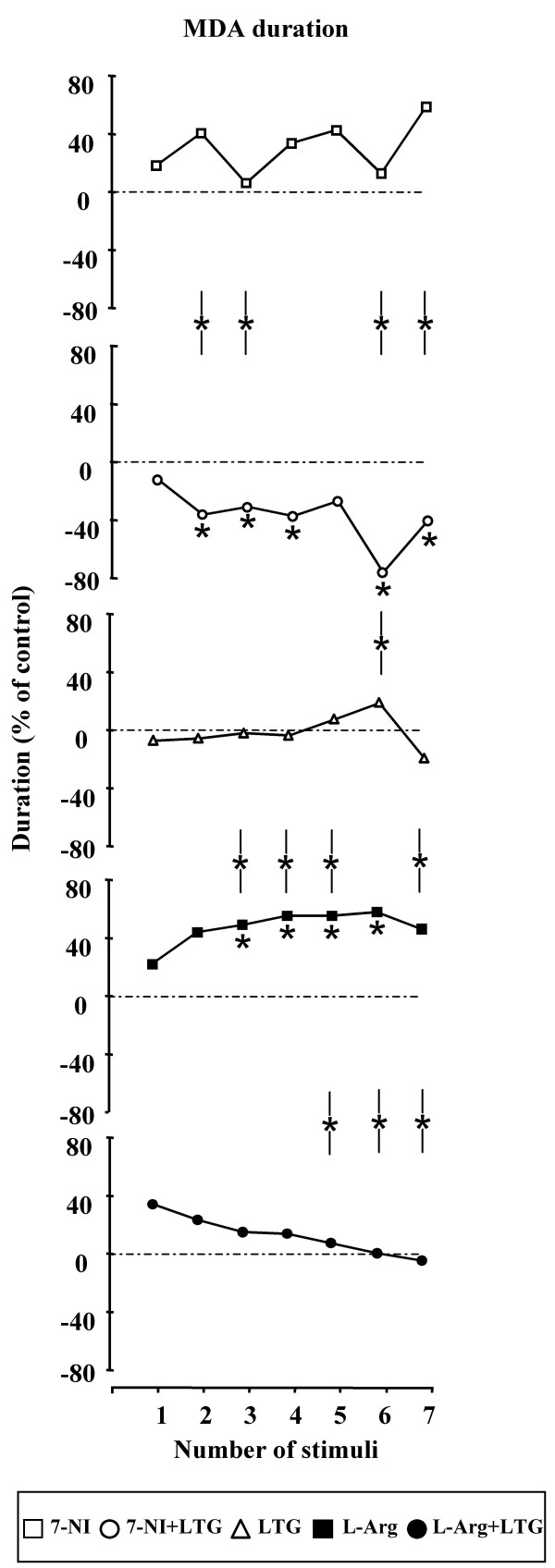
**Effects of 7-Nitroindazole, L-arginine and Lamotrigine administration alone and in combination on MDA duration**. Effects of 7-Nitroindazole (75 mg kg^-1 ^i.p.), L-arginine (1 g kg^-1 ^i.p.) and Lamotrigine (10 mg kg^-1 ^i.p.) administration alone and in combination on MDA duration (n = 10 rats for each treatment). In abscissa the progressive number of train of stimuli applied every 10 min is indicated. Asterisks along graph lines (*) indicate a significant difference versus control values (P < 0.05). Asterisks between lines (-*-) indicate a significant difference between the two treatments (P < 0.05).

**Figure 5:**
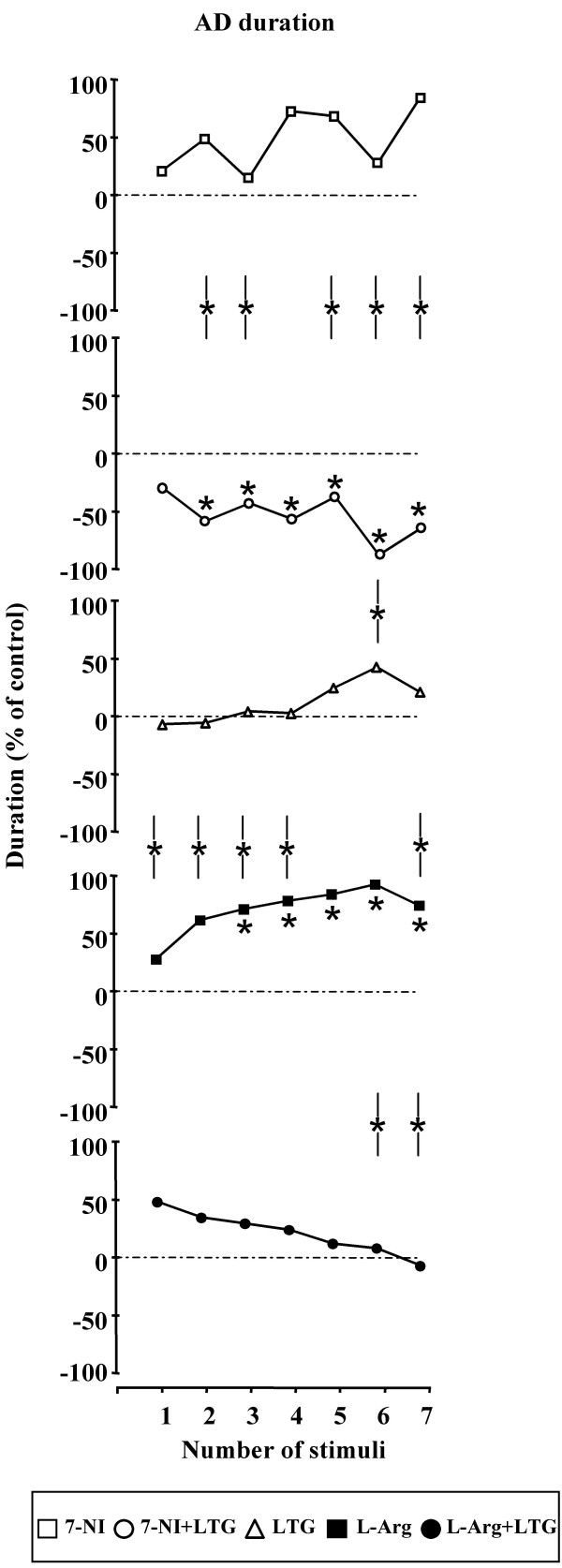
**Effects of 7-Nitroindazole, L-arginine and Lamotrigine administration alone and in combination on AD duration**. Effects of 7-Nitroindazole (75 mg kg^-1 ^i.p.), L-arginine (1 g kg^-1 ^i.p.) and Lamotrigine (10 mg kg^-1 ^i.p.) administration alone and in combination on AD duration (n = 10 rats for each treatment). In abscissa the progressive number of train of stimuli applied every 10 min is indicated. Asterisks along graph lines (*) indicate a significant difference versus control values (P < 0.05). Asterisks between lines (-*-) indicate a significant difference between the two treatments (P < 0.05).

B) MDA and AD durations (Figures 4 and 5 respectively) – 7-NI did not cause significant variations of MDA and AD mean duration. In contrast, the systemic administration of 7-NI and LTG in combination caused a significant decrease of the MDA duration with a maximal inhibitory effect 60 min after drug administration (D%: - 72,14; from 21.54 ± 7.19 sec to 6.00 ± 5.43 sec; P = 0.0015). At the same time, 7-NI and LTG significantly decreased the duration of the AD; in particular, the maximal effect was highlighted 60 min after the drug injection (D%: -86,30; from 16.57 ± 5.55 sec to 2.27 ± 3.08 sec; P < 0.001). The comparative analysis between the treatments showed differences statistically significant between 20^th ^and 70^th ^min. LTG treatment alone failed to induce significant modifications in the two parameters considered. L-arginine induced a progressive increase in the duration of the MDA, between 20^th ^and 70^th ^min after drug administration. In particular, the major efficacy of L-arginine treatment was evidenced 60 min after drug injection (D%: + 58.63; from 17.55 ± 4.91 sec to 27.84 ± 4.67 sec; P < 0.001). Furthermore, the systemic treatment with L-arginine caused, between 20^th ^and 70^th ^min after drug administration, a clear increase of the AD duration, with a maximum at the 60^th ^min (D%: + 93.25; from 12.65 ± 4.33 sec to 24.45 ± 4.10 sec; P < 0.001). The comparative evaluation between LTG and L-arginine treated rats showed significant differences in the duration of MDA and AD particularly between the 30^th ^and 70^th ^min. Furthermore, the systemic co-administration of L-arginine and LTG did not cause any significant modification of MDA and AD durations. The comparison between L-arginine alone and in combination with LTG showed significant differences starting at 50^th ^min after the drug administration for both MDA and AD duration. Comparisons between 7-NI- vs. LTG - and LTG- vs. LTG + L-Arg - induced effects were not significant.

Finally, the evaluation of the evoked responses to a single electrical shock showed significant differences between each pharmacological treatment, paralleling the ones evidenced by MDA parameters, as reported in the table 1.

**Table 1:** Effect of 7-Nitroindazole, L-arginine and LTG administration alone and in combination on the amplitude of evoked responses measured at the maximum of the effect.

Treatment	Control value	Test value	D%	F (_1,18_)	P
7-NI	18.70 ± 2.40	4.10 ± 1.19	-78.00%	295.14	<0.0001
7-NI + LTG	19.1 ± 2.02	1.40 ± 0.51	-92.67%	717.45	<0.0001
LTG	19.40 ± 3.13	1.70 ± 0.82	-91.23%	298.37	<0.0001
L-Arg	19.70 ± 2.66	24.70 ± 3.46	+25.38%	13.06	0.002
L-Arg + LTG	19.1 ± 4.88	13.7 ± 6.66	-28.27%	4.26	0.054

Values are expressed as mean ± standard deviation. Test values are compared to control values. D%: percent difference between test and control values. Control and post-pharmacological treatment values were statistically analysed using an ANOVA test followed by Bonferroni post-hoc test. F: variance ratio. Differences were considered statistically significant when *P *was less than 0.05.

## Discussions and conclusion

The functional involvement of NO in the genesis and the spreading of the seizures as well as in the excitotoxic phenomena has been widely investigated in the last years but clear and definitive conclusions are not still available [27]. In this regard, a lot of experimental researches have demonstrated that NO can act as an anticonvulsant or a pro-convulsant agent depending on the seizure model employed, the type and the dose of drugs used in order to modify cerebral NO levels, the animal strain etc. [28,29]. In fact, NO could exert an anticonvulsant/neuroprotective effect in the induction and/or the propagation of seizures but, at the same time, it could facilitate the seizure maintenance, particularly in the later phases of the epileptic discharge and in the subsequent establishment of the neuronal damage [30-32]. In the context of these discrepant results, it has been showed that NO can activate two different metabolic pathways which could constitute a further basis of the extreme variability of the functional effects reported in the literature. In fact, NO can act on target cells through the classical cGMP cascade, which is responsible for the modulation of different ion channels [33]. On the other hand, more recent experimental observations have revealed that NO is also able to S-nitrosylate the ion channels showing an alternative modality to modulate cell excitability [33]. Furthermore, several experimental and clinical data have also showed a significant increase of nNOS activity in different models of epileptic disorders due to the augmentation of glutamate release and/or the suppression of GABA A receptor activity [18,34]. For all these reasons, NO is considered to have a controversial but, at the same time, a fundamental role in the induction and/or the propagation of the paroxystic activity showing a key functional interplay role between the excitatory glutamate and the inhibitory GABA activities [27,35].

In previous researches, we have highlighted the functional involvement of the nitrergic neurotransmission in the modulatory action of the normal excitability in several neural structures as the hippocampus, the neocortex and different subcortical nuclei in the rat [36-39]. Our experimental data together with pre-clinical and clinical studies have revealed a functional link between NO and hyperactivity phenomena particularly in the hippocampus, which is characterised by an extreme susceptibility to the epileptic seizures [40-42]. Furthermore, recent studies have, also, provided experimental evidences on the potential adjuvant role exerted by manipulating the NO system in the modulation of the anticonvulsant efficacy of several AEDs [15-18]. Among the AEDs, LTG, with a broad therapeutic spectrum on partial epilepsy, shows a co-operative effect with nNOS inhibitors in *in vitro *studies [24] but, in contrast, its anticonvulsant efficacy is not significantly modified by the co-administration with 7-NI in *in vivo *model of epilepsy [22,23]. In the present study, we have explored the possible modulatory role of drugs affecting the NO transmission on LTG anticonvulsant action exerted on the MDA experimental rat model of human partial epilepsy. This model is an example of excitatory re-entrant loop which is involved in the excitatory normal brain function and in the genesis and the spreading of the epileptiform activity as well [25]. In this study we have found that either the preferential nNOS inhibitor, 7-NI, or the anticonvulsant LTG cause a significant reduction of the number of responding animals to AB stimulation. In addition to this effect, the combined treatment with 7-NI and LTG is also able to decrease MDA and AD durations. This additive effect could be due to the combination of the direct inhibition of nNOS induced by 7-NI treatment together with the indirect reduction of NO release following the LTG-induced decrease of glutamate level in the synaptic cleft and the consequent reduction of NMDA receptor activation [43]. On the contrary, L-arginine induces an aggravation of the epileptiform phenomena, as evidenced by the increase of the duration of both MDA and AD, with no influence on the number of responding animals. The not significant effect caused by the treatment with 7-NI or LTG alone on MDA parameters could be due to the significant number of animals which were not responsive to the AB stimulation. This finding constitutes an interesting advance in comparison with our previous experimental data reported in a recent paper which indicate that 7NI, at a lower dose (50 mg kg^-1^), shows a moderate anticonvulsant activity only reducing MDA and AD durations [44]. Otherwise, the co-administration of LTG and L-arginine demonstrates adversative reciprocal effects, indirectly supporting the hypothesis that the increased NO activity could act as pro-convulsant [44,45]. On the other hand, the reversal effect exerted by L-arginine on the LTG-induced reduction of the number of responding animals is likely due to the functional balance between L-arginine-induced increased NO synthesis and LTG-induced reduction of NO levels based on the inhibition of glutamate release [24,43]. Furthermore, the comparative analysis between all the pharmacological treatments, alone or in combination, shows a significant increase of the anticonvulsant effect induced by the combined 7-NI-LTG treatment vs 7NI alone as well as the antagonistic effects induced by L-arginine and LTG. Finally, the DG evoked population spike data, in 7-NI, L-arginine and LTG treated-animals (alone or in combination), revealed modifications in the amplitude strictly consistent with the changes induced in MDA parameters by the same pharmacological tests. In particular, it is possible to highlight the homogeneous response (decrease of the amplitude) caused by the administration of 7-NI and LTG, alone or in combination. On the contrary, the treatment with L-arginine alone caused an increase of the spike amplitude, while under the combined treatment with LTG the spike amplitude was reduced in comparison with control and L-arginine alone and increased with respect to 7-NI and/or LTG-treated animals.

In the context of a suggested NO-mediated pro-convulsant role, our present experimental results are in agreement with other research data which indicate that the nitrergic tone could play a crucial functional role in the proneness to the epileptogenic phenomena also potentially modifying the anticonvulsant efficacy of some antiepileptic drugs [46,47]. On the other hand, the co-operative relationship between 7-NI and LTG suggests an action on a common target, represented by the enhanced glutamate activity; in fact, the increased nNOS activity, strictly related to the type and the severity of seizures, causes an additional release of glutamate in a retrograde or anterograde modality [34,45,48]. 7-NI and LTG could co-operate through several mechanisms at pre- and post-synaptic levels, decreasing the excessive glutamate release and preventing, when administered in combination, the activation of neurotoxic cascade associated with an irreversible brain damage.

It could, therefore, be concluded that the pharmacological manipulation of the nitrergic neuromodulatory system is able to modify the susceptibility and the development of discharge in this experimental model of partial complex seizures. Furthermore, the present study provide electrophysiological evidences for a marked influence of the nitrergic neurotransmission on the anticonvulsant action of LTG. In particular, it could be hypothesised an effect on the overactivity of the excitatory glutamatergic system as evidenced by the enhanced anticonvulsant action of 7-NI and LTG and by the opposite effect due to L-arginine and LTG co-administration. In fact, LTG and 7-NI are both able to inhibit seizure activity in the DG and when co-administered the protective action is significantly potentiated. Although long-term studies are essential before using NO-related drugs to increase the therapeutic action of the AEDs, it is possible to hypothesise a functional interaction between NO inhibitors and AEDs in the inhibitory control of the depolarization and the development of the epileptic activity in the brain.

## Methods

### Animals and surgical procedures

Male Wistar rats, weighing 180–200 g on arrival, were housed at constant temperature of 21°C and a 12 h light/dark cycle, lights on at 8.00 a.m. Thereafter the rats were anaesthetised with urethane (1.2–1.4 g kg^-1 ^intraperitoneally, i.p.). The trachea was cannulated and the skull exposed. The animals were positioned in a stereotaxic apparatus (David Kopf Instruments, Tujunga, CA, U.S.A.) and the body temperature was maintained at 37–38°C using an heating pad. Hearth rate and pupil diameter were monitored during all the experimental session. A craniotomy was performed to expose a wide area of the right cerebral cortex; then the *dura *was reflected. A stimulating depth electrode was placed in the AB on the right side (coaxial bipolar stainless steel electrode: external diameter 0.5 mm; exposed point 25–50 μm) according to the stereotaxic co-ordinates of the Atlas of Paxinos and Watson 1986 [49]. (AB: 1 mm anterior to the interaural line; 3–5 mm dorsal to it and 4.4 mm lateral to the midline). A glass recording electrode, filled with 1% fast Green in 2 M NaCl, was stereotaxically placed in the DG on the right side (DG: 6 mm anterior to the interaural line; 3.0 mm ventral to cortical surface and 1.8 mm lateral to the midline). The animal was grounded through a subcutaneous Ag/AgCl wire in the scapular region. The bioelectric activity of the structure examined was amplified, recorded and printed out on the strip chart of an eight channels polygraph Grass model 7B and then processed by a software package provided by DataWave Technologies (Longmont, CO, U.S.A.).

### Maximal dentate gyrus activation and ictal events identification

In order to obtain stable and repetitive MDA responses and to avoid their progressive increase in the duration, we modified the technique originally described by Stringer and Lothman, 1989 and 1992 [10,25] which employs variable stimulation train durations strictly related to the beginning of the MDA response. In fact, in the present work, 10 sec duration trains of 20 Hz stimuli were given through the AB stimulating electrode. Individual stimuli consisted of 0.3 msec biphasic pulses. The stimulus intensity was initially below that necessary to elicit any response and, then, increased in 100 μA steps until maximal dentate activation occurred (threshold intensity). Furthermore, we used a stimulus intensity 100 μA higher than the threshold intensity for the following stimulations. In every case the stimulation intensity varied from 300 μA to 600 μA. The paroxystic activity induced in the dentate gyrus was simultaneously sent to both a low level DC and a wide band AC pre-amplifiers. MDA was defined by a negative shift of the extracellular potential in DC-coupled recordings as well as by the presence of bursts of population spikes of 20–40 mV in both DC- and AC-coupled traces (Figure 1A). A stimulus train was administered every 2 min until a MDA appeared and then every 10 min for up to 2 hours. Furthermore, single stimuli were given to the ipsilateral AB in order to record evoked potentials in the DG with the aim to compare the amplitude before and after drug administration (Figure 1B).

### Drug treatment

The following chemicals: DMSO, L-arginine and 7-NI were purchased from Sigma Chemical Co. (Sigma, St. Louis, MO, USA). LTG was generously provided by Glaxo-Smith & Kline (Verona, Italy). The experimental study was performed on three groups of animals: in the 1^st ^(controls, n = 10 rats) and 2^nd ^group (vehicle treated, n = 10 rats), the animals were studied for a period of about 120 min in order to verify possible modifications of MDA parameters due to the repetitive stimulations and/or the vehicle treatment. In the 3^rd ^group (treated rats: n = 50, 10 for each single or combined pharmacological treatment), the animals received i.p. injections of 7-NI (75 mg kg^-1^, dissolved in 15% DMSO and made up to final volume by addition of 0.9% NaCl, administered in a volume of 1 ml/100 g body weight) or L-arginine (1 g kg^-1^, dissolved in 0.9% NaCl, injection volume 1–2 ml), and/or LTG, an anticonvulsant able to reduce the glutamate release through the inhibition of voltage-gated Na^+ ^channels (10 mg kg^-1^, dissolved in 30% DMSO and made up to the final volume by addition of 0.9% NaCl). It is noteworthy that 7-NI, L-arginine and LTG show pharmacokinetic and pharmacodynamic profiles compatible with the simultaneous co-administration [50-52]. In fact, 7-NI and L-arginine are capable to significantly modify brain NO levels in a range of 0.5–4.5 hours [53] and LTG is able to cause the reduction of glutamate release in a range of 0.5–4.0 hours [54]. Each pharmacological treatment was performed after five consecutive stable MDA responses (considered as baseline).

### Statistical analysis

For each studied parameter, the data from all animals were averaged (mean ± S.D.) on the basis of the time elapsed from the first stimulation inducing a stable MDA (number of stimulus). The time course of response parameters in control animals was analysed using a one-way repeated measures ANOVA. The last MDA response mean values before drugs administration were considered as the control values for statistical comparisons. Pre- and post-pharmacological treatment parameters (latency, MDA and AD durations, evoked potentials amplitude) were statistically analysed using an ANOVA test followed by Bonferroni post-hoc test. It was not possible to use a repeated measures ANOVA due to the presence of some not responding animals following each drug treatment. Further statistical comparisons were made between different drug treatments (7NI vs 7Ni+LTG or LTG; L-arginine vs L-arginine+LTG or LTG; LTG vs 7-NI or L-arginine). Finally, the chi-square test was used to compare the number of animals responding and not responding to the electrical stimulation after each drug treatment. Differences were considered statistically significant when *P *was less than 0.05.

### Histology

At the end of each experiment, recording and stimulating electrode positions were marked through iontophoretic Fast Green injection and small electrolytic lesion respectively. Then, the animals were killed with an overdose of pentobarbital and perfused with 10% buffered formaline. The brains were removed for histological examination: 30–50 μm serial coronal sections were cut and stained by using Nissl thyonine or Nissl cresyl violet methods, or both.

All animal use procedures were in strict accordance with European Communities Council Directive (86/609/EEC), with the Italian Health Ministry guidelines (D.L. 116/1992) and with Animals Scientific Procedures Act 1986. All efforts were made to minimise the number of animals employed and to reduce their suffering.

## Abbreviations

7-NI; 7-nitroindazole

AB; angular bundle

AD; afterdischarge

AEDs; antiepileptic drugs

cGMP; cyclic guanosine monophosphate

CNS; central nervous system

DG; dentate gyrus,

DMSO; Dimethylsulfoxide

i.p; intraperitoneally

LTG; lamotrigine

MDA; maximal dentate gyrus activation

NMDA; N-methyl-D-aspartate

nNOS; neuronal NOS

NO; nitric oxide

NOS; NO synthase

sGC; soluble guanylyl cyclase

## Authors' contributions

PS and GF equally conceived the study, carried out the electrophysiological study, co-operated in its design, performed the statistical analysis, interpreted the experimental data and edited the manuscript. Both the authors read and approved the final manuscript.
